# Psychosocial health and suicidal ideation among people living with HIV/AIDS: A cross-sectional study in Nanjing, China

**DOI:** 10.1371/journal.pone.0192940

**Published:** 2018-02-22

**Authors:** Wei Wang, Chenchang Xiao, Xing Yao, Yinmei Yang, Hong Yan, Shiyue Li

**Affiliations:** 1 School of Health Sciences, Wuhan University, Wuhan, P.R. China; 2 Department of Epidemiology, University of Florida, Gainesville, FL, United States of America; 3 Wuhan University of Science and Technology City College, Wuhan, P.R. China; University of California at San Diego, UNITED STATES

## Abstract

**Background:**

Suicide is a serious cause of mortality worldwide and is considered as a psychiatric emergency. People living with HIV/AIDS (PLWHA) have higher rates of suicidal behavior than the general population. This study assessed the prevalence and verified the syndemic effect of psychosocial health conditions on suicidal ideation among PLWHA in China.

**Methods:**

An institutional-based cross-sectional study was conducted from July to August 2016 in Nanjing, China, using a self-report questionnaire. Sociodemographic characteristics, infection status, psychosocial variables and suicide ideation reports of participants were collected. Logistic regressions were used to identify potential factors associated with suicidal ideation and to verify the syndemic effect of psychosocial factors. Additionally, odds ratios (ORs) with 95% confidence intervals (95% CI) were computed.

**Results:**

In total, four hundred sixty-five PLWHA participated, 31.6% (n = 147) of whom had suicidal ideation. The results from univariate analysis showed that older age, low education level, being married, having children, and psychosocial variables (high perceived stigma, depression, low self-esteem, social support and resilience) were significantly associated with increased suicidal ideation. Multiple logistic regression models revealed that depression (OR = 2.70, 95%CI = 1.62–4.51), perceived stigma (OR = 1.97, 95%CI = 1.17–3.32), and low social support (OR = 1.85, 95%CI = 1.08–3.20) and self-esteem (OR = 4.11, 95%CI = 2.06–8.16) were statistically significant. PLWHA with at least two psychosocial health problems were nearly 5 times more likely (OR = 4.72, 95% CI 3.11–7.17) to have had suicidal ideation.

**Conclusions:**

Suicidal ideation is frequent among PLWHA in China and is consistent with prevalence estimates from abroad. Psychosocial health problems were the determining factors associated with suicidal ideation, and a syndemic effect of psychosocial health conditions was confirmed in predicting suicidal ideation. Therefore, early screening of high-risk groups for suicidal ideation and more psychosocial health care among PLWHA are needed.

## Introduction

HIV/AIDS is a common health care issue, and there are currently more than 34 million people living with HIV/AIDS (PLWHA) globally [1]. With an economic boom over the past several decades in China, the number of PLWHA continues to increase, and HIV/AIDS has gradually become a serious public health problem in recent years [2]. At the end of December 2015, over 0.58 million PLWHA lived in China, based on statistics from the Chinese Center for Disease Control and Prevention, but as many as 32.1% HIV-positive individuals were not identified in this estimate [3]. Considering that the number of PLWHA continues to grow, the healthcare and management of HIV/AIDS undoubtedly present a major challenge for Chinese health service providers.

Suicide remains an underreported phenomenon in the developing world despite its possible high prevalence, and many studies have reported different suicide rates in several countries and settings [4–7]. Suicidal behavior is a complicated process that ranges in degree of severity, from thinking about killing oneself (i.e., suicidal ideation) to actually doing it (i.e., suicide attempt and completion) [8]. Some studies have reported that suicidal ideation is more common than suicide attempt and completion, and the presence of suicidal ideation increases the risk of suicide attempt and completion [9]. A study of suicidal behavior conducted in 17 Western countries found that in the general population, the lifetime prevalence was 9.2% for suicidal ideation, 3.1% for suicide planning, and 2.7% for suicide attempts, but data varied widely among different countries [10]. In China, a recent community prevalence survey reported a prevalence of 2.3% for suicidal ideation and a prevalence of 1.0% for attempted suicide in general Chinese populations [11].

HIV/AIDS and suicidal behavior have increasingly presented major public health challenges and brought heavy burdens to society. For these reasons, it is important to understand the relationship between HIV infection and suicidal ideation in PLWHA, as well as the role HIV infection plays in the suicidal ideation of PLWHA. Marzuk [12] proposed two hypotheses. The first hypothesis is that HIV infection itself directly leads to suicidal ideation among PLWHA. The second hypothesis is that HIV infection does not increase the risk of suicide; rather, it only plays the role of a catalyst in accelerating the occurrence of suicidal ideation. Consistent with the second hypothesis, HIV/AIDS is a highly stigmatizing chronic disease, and HIV infection is considered to be a traumatic experience and stress-inducing which can lead to negative physical and mental consequences [13, 14]. PLWHA not only suffer from the disease itself but also bear the psychological pressure, economic difficulties and serious social discrimination caused by the disease, which often lead to serious psychosocial health problems, especially related to mental health. Mental health problems can negatively affect treatment, adherence to treatment, and the prognosis of the HIV infection [15, 16]. In recent years, mental health problems have gradually come to the fore as a critical issue among PLWHA, and such problems may cause them to develop suicidal ideation, thus leading to higher mortality. Experts have recognized HIV/AIDS as a potential predictor of suicidal behavior.

It has been reported that suicidal ideation is more common in HIV-positive patients than in the general population [17–19]. However, some studies have not detected a difference [20, 21]. Different studies have revealed that the prevalence of suicidal ideation among people with HIV/AIDS was 31% in London, while the prevalence of suicidal attempt was 23% in France [22, 23]. Studies in China have reported various estimates of the prevalence of suicidal ideation among PLWHA. Lau et al. [24] reported that 34% of HIV-positive individuals who were former blood and/or plasma donors (FBPD) in rural China had suicidal ideation, while another study found that 43.1% of HIV-infected heroin injection drug users (IDUs) in treatment had suicidal ideation [25]. Jin et al.[26] found that 64% of another Chinese cohort of HIV-infected individuals self-reported ever having suicidal thoughts. It is worth considering that the samples investigated in China were small, so we cannot determine whether the prevalence of suicidal ideation among PLWHA in China is consistent with the prevalence estimates from abroad.

In China, it is likely that PLWHA suffer more serious discrimination and lack of available resources than do PLWHA in other countries, so their quality of life is especially poor [27]. For example, data have shown that most PLWHA were infected through homosexual behavior in China [28]. Men who have sex with men (MSM) in China not only suffer physically from the disease but also psychologically due to the stigma against homosexuality [29]. In the traditional Chinese conservative cultural environment, most people do not approve of homosexuality, so MSM have become a highly stigmatized group in Chinese society [30]. Therefore, MSM are more likely to experience various mental health problems [31], including suicidal behaviors, compared to heterosexual individuals. Accordingly, we expected that the prevalence of suicidal ideation among PLWHA is higher in China than prevalence estimates from other countries.

In western countries, various factors, including sociodemographic factors, HIV infection status, sexual behaviors and psychosocial factors, have been reported to be significantly associated with suicidal ideation in PLWHA. For sociodemographic factors, female sex, younger age, low monthly income, low education level and living alone have shown significant associations with suicidal ideation [9, 17, 23, 32–34]. HIV infection status, including high CD4 count, longer infection period, antiretroviral therapy- (ART-) related adverse drug reactions, HIV-related physical symptoms and antiretroviral regimen, also have been reported to be associated with suicidal ideation [9, 17, 22, 35]. Sexual behavior also had significant associations with suicidal ideation in PLWHA, such as homosexual orientation and risky sexual behaviors [36, 37]. For psychosocial factors, suicidal ideation has been significantly associated with low social support, perceived HIV stigma and discrimination, major depression, anxiety, impulsivity and hopelessness [9, 17, 34, 38–40]. These findings are largely from other countries, whereas previous studies in China have suggested that gender, depression, poor social support, and family stresses have been associated with suicidal ideation in Chinese PLWHA [24, 41].

Given the various contributing or protective factors, it may be more reasonable to interpret suicidal ideation among PLWHA using a more holistic perspective, such as the syndemic theory [42], which can explain the magnifying effects of the syndemic of multiple factors, especially psychosocial variables. The term syndemic, which was first used by Singer [43] to describe the inextricable and mutually reinforcing connections among substance use, violence, and AIDS among the urban poor, is defined as several co-occurring and synergistically interacting epidemic factors contributing to the excess of disease in a certain population [44] We hypothesized that there is a syndemic among the psychosocial variables, so it is logical to select several dependable psychosocial scales (depression, self-esteem, resilience, perceived stigma and social support) which may help in the early detection of those at risk of suicidal ideation among PLWHA.

The first purpose of this Chinese study was to describe the prevalence of suicidal ideation in PLWHA and determine whether it is consistent with prevalence estimates from abroad. The second purpose was to further explore factors associated individually with suicidal ideation among PLWHA in China, as well as the relationship between multiple psychosocial variables and suicidal ideation, by applying syndemic theory.

## Method

### Participants and eligibility criteria

This cross-sectional study was conducted in Nanjing, Jiangsu Province, China, from July to August in 2016 among PLWHA in the Second Affiliated Hospital of Southeast University. HIV-positive individuals were eligible to participate if they had been diagnosed and registered with the Chinese National Information System for AIDS Prevention and Control (CNISAPC), which is the official entry point for HIV/AIDS patients to receive regular follow-up and health care according to national guidelines.

### Procedure

Our survey workers carried out anonymous face-to-face interviews with the participants in a separate room in order to protect the privacy of the respondents. Interviewers had received enough training before the interviews and had several in-person reviews throughout the entire study. The training also included quality control strategies, such as reexamining and investigating the questionnaires and settling issues that might occur during the fieldwork.

All individuals included in the current study were able to provide their written informed consent and each received a 50 ¥ (approximately US$8) supermarket voucher for study participation. Participants were also advised that they could withdraw from the study at any time. This study was approved by the hospital’s ethics committee and by the Research Ethics Committee of the Graduate School of Medicine at Wuhan University.

### Measures

#### Sociodemographic characteristics

The baseline characteristics of participating PLWHA included age, sex, education level, marital status, employment status, monthly income, place of residence (rural, urban) and sexual orientation. Family factors included whether participants had children, their current household composition and the status of HIV disclosure to family members.

#### HIV/AIDS infection characteristics

Data were collected by interviewing the participants and reviewing their medical records. HIV/AIDS infection condition included CD4 level, route of infection, duration of infection, duration of highly active antiretroviral therapy (HAART) and whether their HIV/AIDS diagnosis was combined with other sexually transmitted diseases (STDs).

#### Psychosocial variables

Depression was measured with the Center for Epidemiological Studies Depression (CES-D) scale which has 20 items [45]. Participants were asked how often they had experienced depressive symptoms in the past week. Items were answered on a 4-point scale ranging from 0 (less than a day or never) to 3 (5–7 days). The total score in this study ranged from 0 to 60, with a cut-off score of 16 indicating significant depressive symptoms [46] (Cronbach's alpha = **0.914 in this study**).

Self-esteem was measured by the Rosenberg Self-Esteem Scale [47] was used to assess participants' global feelings of self-worth. The scale was comprised of five positively worded and five negatively worded items. Participants were asked to rate the extent to which they agreed or disagreed with each of the items, and items were answered on a 4-point scale ranging from 0 (strongly disagree) to 3 (strongly agree). Positively worded responses were reverse-scored, so that when the total scores were calculated, a higher score indicated higher self-esteem, whereas a lower score (<25) indicated lower self-esteem [48] (Cronbach's alpha = **0.905 in this study**).

Resilience was measured with the 14-item Ego-Resilience scale (ER89) which developed by Block and Kremen [49] and based on their experience with earlier resilience scales. The focus of the ER89 is primarily flexibility, curiosity, generosity and social skills. Participants were asked to indicate the degree to which they agreed with each of the items, based on a 4-point scale ranging from 0 (does not apply at all) to 3 (applies very strongly). We defined the group of significantly lower resilience as those with a total score lower than 35, which was the 25th percentile in our study (Cronbach's alpha = **0.851 in this study**).

Perceived Stigma was measured by the HIV Stigma-Revision Scale[50]. With each item, participants were required to rate their perceived stigma experience using a scale of 1 (strongly disagree) to 4 (strongly agree). A total score was calculated by computing the sum of the 30 items. The perceived stigma scale contains four domains: anticipated stigma (9 items), negative self-image (7 items), concern with public attitude (6 items) and disclosure concerns (8 items). Higher scores indicated a greater perceived stigma experience. We used the 75th percentile score of 88 as the cut-off to define the group having a problem with stigma (Cronbach's alpha = **0.923 in this study**).

Social support was measured with.the Social Support Rating Scale (SSRS)which originally developed in Chinese by Xiao [51], has already been widely used in different Chinese communities and shown to be valid and reliable[52]. The SSRS contains 10 items and evaluates social support in the following three domains: subjective support (4 items), objective support (3 items), and support usage (3 items). Subjective support reflects the perceived interpersonal network on which an individual can depend. Objective support reflects the degree of actual support received in the past. Support usage refers to the pattern of behavior that an individual utilizes when seeking social support [52]. Higher scores indicate stronger social support, and we defined those with a total score lower than 30 to be the low-level social support group (Cronbach's alpha = **0.790 in this study**).

#### Suicidality

Suicidality was measured with three items. Participants were asked if they had suicidal ideation after HIV diagnosis. Participants who reported having suicidal ideation were then asked whether they had suicidal plans and suicidal attempts.

The question on suicidal ideation was as follows: “Have you ever had suicidal ideation after HIV diagnosis? (0 = no, 1 = yes)”. The subsequent item for suicide plan was as follows: “Have you ever had a plan of taking your own life after HIV diagnosis? (0 = no, 1 = yes)”. Finally, suicide attempts were assessed with the following item: “Have you ever attempted to take your own life after HIV diagnosis (0 = no, 1 = yes), and if so, how many times? (0 = 0 times, 1 = 1 times, 2 = 2 or more times)”.

### Statistical analysis

Data were double-entered by EpiData 3.1 (The EpiData Association, Odense, Denmark) software. Baseline descriptive statistics were calculated to summarize sociodemographic characteristics, suicidal ideation, infection status, and psychosocial variables. The categorical variables were described using frequencies and percentages. Univariate analysis using chi-square tests and univariate logistic regression for categorical variables was performed to detect the association between potential risk factors and suicidal ideation. In the syndemic effect stage, a syndemic count variable was created by counting the number of psychosocial health problems of each participant and allocating participants into different groups based on the number of their syndemic variables.

Different suicidal ideation rates were assessed by a multivariate logistic regression model using the variables that were at least marginally significant, with p<0.10 in the univariate analysis, and other significant factors according to previously published studies. Model (1) consisted of only sociodemographic characteristics. Model (2) also included family factors. We combined sociodemographic characteristics, family factors and infection status in Model (3). Then, we added psychological variables and social variables in Models (4) and (5), respectively. To evaluate goodness of fit, we included the average Nagelkerke’s R2 for each model. All statistical tests were 2-sided, evaluated as significant at the p<0.05 level, and we performed statistical analysis using the Statistical Package for Social Sciences (version 21.0 for Windows; IBM SPSS Statistics, Armonk, NY, USA).

## Results

### Participant sociodemographic characteristics

A total of 465 HIV-positive individuals were interviewed, and approximately 2% of people approached declined to complete the interview. The average of age was 37.22±12.01 years, and ages ranged from 18 to 77 years, with 71.1% of the participants being younger than 45 years old. Among the participants, 95.1% (n = 442) were males and 4.9% (n = 23) were females, 53.7% reported never having been married and 13.8% were divorced or widowed. Approximately 34.0% of the participants reported monthly incomes lower than 3000 RMB (approximately US$440), while 34.2% reported monthly incomes higher than 5000RMB (approximately US$740). Most of them were employed (67.3%), with a college degree or higher level of education (60.4%) and with a registered residence in an urban area (63.7%). Additionally, 50.3% of the participants identified as homosexual. About forty percent of individuals had children and 51.8% were living with family members; the majority (70.5%) of those living with family members had disclosed their HIV infection to family members. The basic sociodemographic information of the PLWHA is summarized in **Table 1**.

**Table 1:** Socio-demographic characteristics by suicidal ideation status (n = 465).

Characteristics	Number in study	Suicidal ideation	Univariate analysis	
	n (row %)	n (row %)	OR	95% CI
Age (years)				
<25	62 (13.3)	20 (32.3)	1.33	0.73–2.41
25–44	269 (57.8)	71 (26.4)	1.00	
>45	134 (28.9)	56 (41.8)	2.00	1.29–3.10**
Gender				
Male	442 (95.1)	139 (31.4)	1.00	
Female	23 (4.9)	8 (34.8)	1.16	0.48–2.81
Residence				
Urban	296 (63.7)	88 (29.7)	1.00	
Rural	169 (36.3)	59 (34.9)	1.27	0.85–1.90
Educational level				
High school or lower	184 (39.6)	70 (38.0)	1.80	1.05–3.07*
Junior college	102 (21.9)	26 (25.5)	1.00	
University or higher	179 (38.5)	51 (28.5)	1.17	0.67–2.02
Marital status				
Never married	250 (53.7)	63 (25.2)	1.00	
Married	151 (32.5)	63 (41.7)	2.13	1.38–3.27**
Divorced/Widowed	64 (13.8)	21 (32.8)	1.45	0.80–2.63
Employment status				
Employed	313 (67.3)	97 (31.0)	1.00	
Unemployed/retired/students	152 (32.7)	50 (32.9)	1.09	0.72–1.65
Monthly income (RMB)				
<3000	158 (34.0)	51 (32.3)	1.21	0.75–1.95
3000–5000	148 (31.8)	51 (34.5)	1.33	0.82–2.16
>5000	159 (34.2)	45 (28.3)	1.00	
Sexual orientation				
Homosexual	234 (50.3)	68 (29.1)	1.00	
Heterosexual	117 (25.2)	37 (17.5)	1.13	0.70–1.83
Bisexual/undecided	114 (24.5)	42 (36.8)	1.42	0.89–2.29
Having chidren				
Yes	188 (40.4)	76 (40.4)	1.97	1.32–2.93*
No	277 (59.6)	71 (25.6)	1.00	
Living situation				
Alone	137 (29.5)	43 (31.4)	0.84	0.54–1.31
Friends or others	87 (18.7)	19 (21.8)	0.51	0.29–0.91
Family members	241 (51.8)	85 (35.3)	1.00	
HIV disclosure to family members				
Yes	328 (70.5)	103 (31.4)	1.00	
NO	137 (29.5)	44 (32.1)	1.03	0.67–1.59

*P<0.05.

** P<0.01

### HIV/AIDS infection characteristics

Three hundred forty (73.1%) participants were infected by homosexual contact, 78.9% of the respondents were diagnosed with HIV more than 12 months before the time of data collection, 320 (69.1%) of respondents had a CD4 level lower than 500, most of the respondents (67.7%) had been on HAART for longer than 12 months and approximately 14.0% of the participants had complications of other STDs The characteristics of HIV/AIDS infection of the PLWHA are shown in Tab**le 2**.

**Table 2:** HIV/AIDS infection characteristics by suicidal ideation status.

Characteristics		Number in study	Suicidal ideation	Univariate analysis	
		n (%)	n (row%)	OR	95% CI
Route of infection					
Homosexual contact		340 (73.1)	102 (30.0)	1.00	
Heterosexual contact		98 (21.1)	36 (36.7)	1.36	0.85–2.17
Other		27 (5.8)	9 (33.3)	1.17	0.51–2.68
Duration of infection					
<12 months		98 (21.1)	26 (26.5)	1.00	
≥12 months		367 (78.9)	121 (33.0)	1.36	0.83–2.42
Duration of HAART					
<12 months		150 (32.3)	44 (29.3)	1.00	
≥12 months		315 (67.7)	103 (32.7)	1.17	0.77–1.79
CD4 level					
≤500		320 (69.1)	98 (30.6)	1.00	
>500		143 (30.9)	47 (32.9)	1.11	0.73–1.69
Other STDs					
Yes		65 (14.0)	22 (33.8)	1.13	0.65–1.96
No		400 (86.0)	125 (31.3)	1.00	

HARRT = Highly active antiretroviral therapy; STDs = Sexually transmitted diseases

### Prevalence of suicidal ideation

Most participants had never been suicidal; however, one hundred forty-seven (31.6%; n = 147) participants had suicidal ideation after HIV diagnosis. Fifty-three people (11.4% of the total sample) had a suicide plan, and among them, 22.6% (n = 12) had made a suicide attempt after HIV diagnosis. The rates of suicidal ideation among PLWHA with different sociodemographic characteristics are presented in **Table 1**.

### Psychosocial status among PLWHA

**Table 3** depicts the psychosocial health conditions among the participating PLWHA. Of the participants, 29.0% fell in the group of low social support, 15.3% in the group of low self-esteem and 29.9% in the group characterized by low levels of resilience. The prevalence of significant depression was 38.1%, and 117 (25.2%) experienced severe perceived stigma.

**Table 3:** Psychosocial variables by suicidal ideation status.

Characteristics	Number in study	Suicidal ideation	Univariate analysis	
	n (%)	n (row %)	OR	95% CI
Depression				
Low level (score <16)	288 (61.9)	57 (19.8)	1.00	
High level (score ≥16)	177 (38.1)	90 (50.8)	4.19	2.77–6.34***
Social support				
Low level (score ≤30)	135 (29.0)	61 (45.2)	2.34	1.54–3.56***
High level (score >30)	330 (71.0)	86 (26.1)	1.00	
Perceived stigma				
Low level (score <88)	348 (74.8)	88 (25.3)	1.00	
High level (score ≥88)	117 (25.2)	59 (50.4)	3.01	1.94–4.65***
Self-esteem				
Low level (score ≤25)	71 (15.3)	51 (71.8)	7.92	4.50–13.94***
High level (score >25)	394 (84.7)	96 (24.3)	1.00	
Resilience				
Low level (score ≤35)	139 (29.9)	55 (39.6)	1.67	1.10–2.53***
High level (score >35)	326 (70.1)	92 (28.2)	1.00	

***P<0.001

### Potential factors associated with suicidal ideation

In the univariate analysis, four sociodemographic variables (age, education level, marital status, whether participants had children) were significant in relation to suicidal ideation among PLWHA (p<0.05). The older-aged group (>25 years of age) were over 2 times more likely (OR = 2.00, 95%CI = 1.29–3.10) to have suicidal ideation compared with the younger- and middle-aged group (25–44 years of age). Having a high school or lower education level was predictive of greater vulnerability to suicidal ideation (OR = 1.80, 95%CI = 1.05–3.07) compared with the junior college group. Married participants (OR = 2.13, 95%CI = 1.38–3.27) demonstrated a substantial increase in suicidal ideation compared with never-married participants. Participants with children were also at higher risk of thinking about suicide (OR = 1.97, 95%CI = 1.32–2.93). Sex, residence, educational level, employment status, monthly income, sexual orientation, living situation, and HIV disclosure to family members were not significantly associated with suicidal ideation (**Table 1**). All clinical characteristics were found had no significant association with suicidal ideation (**Table 2**). The results from the univariate analysis among psychosocial variables indicated that PLWHA who perceived severe stigma (OR = 3.01, 95% CI = 1.94–4.65); possessed higher levels of depressive symptoms (OR = 4.19, 95%CI = 2.77–6.34); and had low levels of social support (OR = 2.34, 95% CI = 1.54–3.56), self-esteem (OR = 7.92, 95%CI = 4.50–13.94) and resilience (OR = 1.67, 95%CI = 1.10–2.53) were at increased risk of suicidal ideation (**Table 3**).

### Factors associated with suicidal ideation in multivariate analysis

**Table 4** presents results from the multiple logistic regression models. Factors significantly associated with suicidal ideation in univariate analyses (age, education level, marital status, with or without children, social support, self-esteem, resilience, perceived stigma and depression) were included in these models. In addition, we also examined sex, employment status, CD4 count and duration of HAART as candidate variables in the model analysis, according to the findings of several previous studies [53–55]. Model (1) showed that among PLWHA, being married was associated with increased suicidal ideation (OR = 1.92, 95%CI = 1.08–3.40) compared with the never-married group. Model (4) demonstrated that high levels of depression (OR = 3.19, 95%CI = 1.93–5.26) and low levels of self-esteem (OR = 4.77, 95%CI = 2.44–9.35) were significantly associated with increased odds of having suicidal ideation. When perceived stigma and social support were combined in Model (5), the effects of depression and self-esteem remained significant (ORs = 2.70 and 4.11, respectively). In this model, all candidate variables were included, and the Nagelkerke’s R2 simultaneously rose to 0.290, indicating a relatively good fit for the model. The effects of marital status were no longer significant, and experiencing severe perceived stigma (OR = 1.97, 95% CI = 1.17–3.32) and having low levels of social support (OR = 1.85, 95%CI = 1.08–3.20) were significantly associated with higher odds of having suicidal ideation.

**Table 4:** Association between suicidal ideation and demographics, family factors, HIV/AIDS infection status, and psychosocial variables (presented as odds ratios (OR)) (n = 465).

Characteristics	Model (1)	Model (2)	Model (3)	Model (4)	Model (5)
	OR(95%*CI*)	OR(95%*CI*)	OR(95%*CI*)	OR(95%*CI*)	OR(95%*CI*)
**Sociodemographics**					
Age (years)					
<25	**Reference**				
25–44	0.58(0.31,1.11)	0.53(0.27,1.03)	0.53(0.27,1.02)	0.55(0.27,1.14)	0.58(0.28,1.23)
>45	0.79(0.34,1.81)	0.67(0.29,1.59)	0.69(0.29,1.64)	0.95(0.37,2.47)	1.04(0.39,2.77)
Sex					
Male	**Reference**				
Female	0.79(0.31,1.99)	0.79(0.31,2.00)	0.77(0.30,2.00)	1.09(0.39,3.05)	1.03(0.35,2.98)
Educational level					
High school or lower	1.20(0.71,2.02)	1.18(0.70,2.00)	1.18(0.70,2.00)	0.81(0.45,1.45)	0.82(0.45,1.49)
Junior college	0.78(0.45,1.38)	0.76(0.43,1.34)	0.75(0.42,1.33)	0.60(0.32,1.11)	0.56(0.30,1.07)
University or higher	**Reference**				
Employment status					
Employed	**Reference**				
Unemployed/retired/students	1.01(0.65,1.55)	1.01(0.65,1.57)	1.03(0.66,1.60)	0.75(0.46,1.23)	0.72(0.43,1.20)
Marital status					
Never married	**Reference**				
Married	1.92(1.08,3.40) *	1.57(0.70,3.52)	1.57(0.70,3.54)	1.47(0.61,3.53)	1.36(0.56,3.28)
Divorced/Widowed	1.32(0.66,2.64)	1.11(0.51,2.46)	1.11(0.50,2.46)	1.08(0.46,2.56)	0.80(0.33,1.94)
**Family factors**					
Had Chidren					
Yes		**Reference**			
No		0.81(0.38,1.68)	0.82(0.39,1.72)	0.79(0.36,1.77)	0.70(0.31,1.59)
Living situation					
Alone		1.12(0.68,1.85)	1.11(0.68,1.85)	1.05(0.61,182)	0.89(0.50,1.59)
Friends or others		0.66(0.35,1.26)	0.66(0.35,1.26)	0.76(0.38,1.55)	0.82(0.40,1.68)
Family members		**Reference**			
**HIV/ADIS infection status**					
Duration of HAART					
<12 months			0.99(0.62,1.57)	0.93(0.56,1.55)	0.97(0.58,1.63)
≥12 months			**Reference**		
CD4 level					
≤500			0.86(0.54,1.35)	0.95(0.57,1.58)	0.95(0.56,1.62)
>500			**Reference**		
**Psychosocial variables**					
Depression (reference = low)				3.19(1.93,5.26) ***	2.70(1.62,4.51) ***
Resilience (reference = high)				0.77(0.46,1.31)	0.67(0.39,1.16)
Self-esteem (reference = high)				4.77(2.44,9.35) ***	4.11(2.06,8.16) ***
Perceived stigma (reference = low)					1.97(1.17,3.32) *
Social support (reference = high)					1.85(1.08,3.20)*
**Nagelkerke’s R2**	0.053	0.061	0.062	0.258	0.29

Model(1): sociodemographic

Model(2): sociodemographic + Family factors

Model(3): sociodemographic + Family factors + HIV/AIDS infection status

Model(4): sociodemographic + Family factors + HIV/AIDS infection status + Psychological variables

Model(5): sociodemographic + Family factors + HIV/AIDS infection status + Psychosocial variables

* P<0.05

***P<0.001

### Syndemic effect of psychosocial variables

The results of the final syndemic analysis are presented in **Table 5**. Of the participants, 38.3% suffered from a psychosocial syndemic (i.e., having two or more psychosocial problems). It was found that a psychosocial syndemic had a magnifying effect on suicidal ideation (OR = 4.72, 95% CI 3.11–7.17). When using the number of syndemic factors to divide into three groups, the low-level syndemic (having two or three psychosocial health problems) and high-level syndemic (having four or five psychosocial health problems) groups were nearly 4 and 9 times more likely, respectively, to have had suicidal ideation than the non-syndemic group (OR = 3.83, 95% CI 2.44–6.01; OR = 9.25, 95% CI 4.59–18.62, respectively).

**Table 5:** Associations between the number of syndemic conditions and suicidal ideation among PLWHA.

	Number in study	Suicidal ideation	Univariate analysis	
	n (%)	n (row %)	OR	95% CI
Have a syndemic				
No(Have no more than one psychosocial problems)	287 (61.7)	54 (18.8)	1.00	
Yes (Have two or more psychosocial problems)	178 (38.3)	93 (52.2)	4.72	3.11–7.17***
Number of syndemic conditions				
No (Have no more than one psychosocial problems)	287 (61.7)	54 (18.8)	1.00	
Low (Have two to three psychosocial problems)	134 (28.8)	63 (47.0)	3.83	2.44–6.01***
High (Have four to five psychosocial problems)	44 (9.5)	30 (68.2)	9.25	4.59–18.62***

***P<0.001

## Discussion

Suicide and HIV/AIDS remain two of the greatest healthcare issues, especially in low- and middle-income countries [56]. According to the World Health Organization (WHO), more than 800,000 people die globally each year by committing suicide [57]. Likewise, global HIV/AIDS trends show that the number of PLWHA has increased rapidly. In the present survey, we found that 31.6% (147 out of 465) of PLWHA had ever had suicide ideation after they were diagnosed with HIV infection, and 12 (2.6%) of these respondents had actually attempted suicide at least once. Apparently, the prevalence of suicidal ideation and attempts among PLWHA are significantly higher than those in the general Chinese population [11,24–26]. The prevalence estimates of suicidal ideation among PLWHA in this study are in line with those found in studies conducted in Great Britian (31%) [22], Ethiopia (33.6%) [53], but lower than in prior Chinese studies [24–26]. Similarly, the study revealed that the prevalence of suicidal attempts among HIV-positive patients was consistent with findings from other studies in Uganda (3.1%) [58]. These findings suggest that the prevalence of suicidal ideation in China is consistent with that of other countries. Although it is not consistent with our hypothesis, there is also an urgent need to increase psychological counseling services and early screening for suicidal ideation among PLWHA in China.

In this study, marital status was significantly associated with suicidal ideation. The married group was 2.1 times more likely to develop suicidal ideation than individuals who were never married. The cause(s) of this finding are likely to be complicated, as is the case for age that was also found to be significantly associated with suicidal ideation. The older-aged group was over two times more likely to have suicidal ideation than the younger- and middle-aged group. Most of the participants in this study identified as homosexual or bisexual, and their ages at marriage were relatively higher. Recent studies have shown that MSM usually delay marriage, compared to the general Chinese population. However, different results have been obtained by some studies from other countries, where patients who were single were more likely to develop suicidal ideation than patients who were married [59, 60]. We speculate that married PLWHA in China feel more social and family pressure; additionally, the different cultural context may explain the increased suicidal ideation. Our participants with high school or lower education levels demonstrated more vulnerability to suicidal ideation than those who had attended junior college and university or higher. The findings from this study further indicated that those who had children more frequently had suicidal ideation. HIV-affected children frequently experience loss of acceptance and support due to suffering from stigmatization and they may tend to have a negative attitude toward their future development [61]. Therefore a possible reason why our participants with children had suicidal ideation is that they worry that their children would be discriminated against by others and the children’s future would be influenced by their HIV infection.

Among the central findings of this study, psychosocial factors, including depression, stigma, low levels of social support and low self-esteem, were associated with the high occurrence of suicidal ideation. These results are consistent with those of previous studies among PLWHA [17, 28, 34, 38]. HIV-related stigma mainly refers to prejudice and discrimination directed at individuals perceived to have HIV or AIDS. In our study, 25.2% of PLWHA had experienced severe HIV-related stigma, and similarly, a recent study reported that 27.1% of participants experienced such stigma [62]. Previous research has established a link between stigma attributed to negative mental health outcomes, including low self-esteem, low levels of social support, anxiety and depression [63, 64]. Those negative effects of stigma may aggravate the psychological pressure of PLWHA, which may then lead to suicidal ideation, because they may consider suicide a better choice for ending their physical and emotional pain and the discrimination resulting from the disease.

In addition, participants who had suicidal ideation reported significant depression symptoms more frequently. Depression is a common mental disorder that presents with not only worse mood, but also reduced interest, energy, sleep, appetite, and concentration. Significant depressive symptoms are often found with long-term chronic diseases and can result in increased risk of suicide. Similarly, in studies outside of China, it has been reported that depression was more prevalent in HIV-positive individuals than in the general population [65]. The prevalence of severe depression was reported to be 12% among PLWHA in India [62]. Previous studies have also indicated an association between depressive symptoms and suicidal ideation among PLWHA [26, 65]. Therefore, the prevention and treatment of depression play an important role in reducing suicidal ideation. Unfortunately, psychiatric services are a major weakness in most regions in China. PLWHA can utilize psychological or psychiatric services only in towns or cities, and such services are unlikely to be affordable to many rural PLWHA. Therefore, increased and more widely available services for psychological problems among PLWHA are desperately needed.

Lack of enough social support was also viewed as a risk factor of having suicidal ideation in the current study. HIV/AIDS is a chronic illness that can be accompanied by psychiatric problems, which brings a heavy burden to society and families. Having enough social resources generally buffers against adverse psychological responses to stressful situations [66]; thus, high levels of social support may have a moderating effect on psychiatric problems. Similarly, suicide risk may be reduced by even the mere perceived availability of social support, and this perceived social support also could have a positive influence on health and mood [66, 67]. In serious illnesses, higher levels of social support tend to be associated with lower levels of depression [68, 69]; similarly, low levels of social support are associated with increased suicide risk in some patients with chronic disease [70]. Likewise, social support plays an important role in the psychological adjustment of PLWHA, and there is a strong inverse relationship between high-level social support and depression in PLWHA [71, 72]. One study conducted in Nepal found that those reporting suicidal ideation also reported low family support, with this complex mechanism as a major correlate of depression symptoms among PLWHA [55]. Therefore, enhancing family counseling and support services may be beneficial in decreasing suicidal ideation among PLWHA and improving their mental health and overall wellness.

Additionally, self-esteem is a major contributor to risk for suicidal ideation. One previous study has shown that stigma is associated with decreased self-esteem among people with mental health problems [73]. As expected, in the present study, low self-esteem was significantly associated with increased suicidal ideation among PLWHA. A recent study reported that, based on the results of a univariate analysis, HIV-positive MSM with low levels of self-esteem were at increased risk of reporting suicidal ideation; however, this relationship was non-significant in multivariable logistic regression models [28].These results suggested that we should pay more attention to PLWHA with low self-esteem.

The most important finding to note in this study is that the results of the final syndemic analysis showed that the psychosocial syndemic revealed a magnifying effect in predicting suicidal ideation. A higher number of psychosocial health problems were associated with greater suicidal ideation among PLWHA, with the effect more prominent when all problems were included. The biggest advantage of our study was the integration of various psychosocial health problems to form a comprehensive framework for the preliminary evaluation of suicidal ideation. To some extent, our study also has other advantages compared with related preexisting studies. First, to our knowledge, this is the first study with such a large sample, examining comprehensive psychosocial predictors of suicidal ideation among PLWHA in China. Additionally, the results mainly support our previous hypotheses and expectations and have significant public health policy implications, as well as provide robust evidence for improving health service guidelines.

Several limitations of our study should also be noted. First, there was no non-HIV- positive comparison group in the current study. Examples of comparison groups that might be of interest include (1) demographically comparable people from the Nanjing general population, and (2) adults from that population who have different life-threatening diseases (e.g., cancer). Second, the present study uses a cross-sectional study design, which makes it difficult to determine the direction of the causality between the risk factors and suicidal ideation. Third, our sample is not representative of the full population of PLWHA in China, and there is a gender imbalance in our study. Fourth, all data were measured by self-report, which means that there may be biases in our study data. Finally, suicidal ideation and suicide attempts were not assessed using a mature scale; thus, using a more scientific measurement, such as the Columbia Suicide Severity Rating Scale [74], could show greater reliability.

In conclusion, suicidal ideation is highly prevalent among PLWHA in China, which is consistent with findings from abroad. Our study successfully confirmed the syndemic effect of psychosocial health conditions to predict suicidal ideation among PLWHA, with the factors contributing to suicidal ideation including depression, stigma, low self-esteem and poor social support. This suggests that a more integrated intervention in a combination of psychological, behavioral, and social aspects is needed to address the syndemic conditions. Also, screening, treatment, suicide education and referral services for suicidal individuals are essential. In addition, reducing HIV related stigma, and strengthening the self-esteem and social support of PLWHA, is constructive in mediating the relations between risk factors and suicide.

## Supporting information

S1 Database Database for all variables. (XLS)
